# Neuropsychological tests used for dementia assessment in Japan: Current status

**DOI:** 10.1111/ggi.14678

**Published:** 2023-09-25

**Authors:** Shinichiro Maeshima, Aiko Osawa, Koki Kawamura, Takako Yoshimura, Eri Otaka, Yayoi Sato, Ikue Ueda, Naoki Itoh, Izumi Kondo, Hidenori Arai

**Affiliations:** ^1^ National Center for Geriatrics and Gerontology Obu Japan

**Keywords:** activities of daily living, caregiver burden, dementia, questionnaire, survey

## Abstract

**Aim:**

This study aimed to investigate the assessment tools dementia specialists use in clinical practice, reasons for their use and assessment‐related factors.

**Methods:**

A questionnaire survey was carried out from 15 September 2021 to 20 October 2021 among 1858 dementia specialists in Japan, with responses obtained via mail or using a Web form accessed via a Web address.

**Results:**

Of the 1858 specialists who were sent the questionnaire, 574 responded, yielding a response rate of 32.2%. Almost all respondents stated that the main purposes of neuropsychological testing were to identify the pathophysiology and aid diagnosis. Most respondents identified behavioral and psychological symptoms of dementia as important factors for assessment. The most commonly used tests were the Hasegawa Dementia Scale‐Revised and Mini‐Mental State Examination, often used as screening tools. The Mini‐Mental State Examination, Clock Drawing Test and Cube Copying Test were common assessments carried out directly by specialists. Quality of life and burden of care were less commonly assessed.

**Conclusions:**

Despite the main purpose of carrying out neuropsychological tests on dementia patients is to “understand the pathophysiology” and “aid in diagnosis,” many assessment methods were chosen as screening methods carried out in a short time during clinic hours. The lack of evaluation of care burden and QOL, considered important by specialists, is an issue for the future in treating people with dementia, a life disability. **Geriatr Gerontol Int 2024; 24: 102–109**.

## Graphical abstract

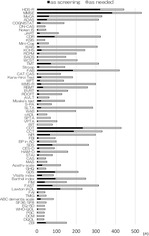

## Introduction

Global population aging has led to an increase in the number of patients with age‐related neurodegenerative diseases, including dementia.^1^ Dementia is characterized by cognitive decline and significantly affects daily life, making its management challenging for patients, their families and society. Therefore, early diagnosis and appropriate assessment for selecting treatment and care have become increasingly important.^2^, ^3^

Neuropsychological testing is vital for diagnosing and assessing dementia.^4^ It helps to identify abnormal cognitive patterns and dysfunctions, providing an objective and in‐depth assessment of a patient's condition. This contributes to accurate diagnosis, post‐diagnosis support, treatment and care planning, and evaluation of intervention effectiveness.^5^ Periodic neuropsychological testing can help to identify changes in cognitive function, predict prognosis, evaluate treatment efficacy, plan interventions and guide treatment modification as necessary.

Various neuropsychological tests and clinical assessments have been proven useful in patients with dementia. These assessments are used for diagnosing dementia, assessing its severity, evaluating cognitive functions and ability to carry out activities of daily living (ADLs), assessing behavioral and psychological symptoms of dementia, and evaluating caregiver burden.^6^, ^7^ However, their use is not regulated, and the actual utilization of neuropsychological testing in dementia care by specialists remains unclear.^8^

Therefore, we carried out a questionnaire survey among dementia specialists to investigate the assessment tools they use in clinical practice, reasons for their use and assessment‐related factors (assessors, assessment timing and time required for each assessment). This survey, the first of its kind carried out among dementia specialists, can help to improve dementia treatment and care.

## Methods

We mailed a request letter and questionnaire to 1858 eligible specialists. We obtained lists of dementia specialists from the Japanese Society for Dementia Research and Japanese Psychogeriatric Society, and removed duplicate names. The respondents replied via mail or using a Web form accessed via a web address. A reminder letter was sent after 2 weeks, and the survey period lasted from 15 September 2021 to 20 October 2021.

The survey collected information on the respondents' basic demographic characteristics (the department and type of hospital they worked at), experience in dementia care, time required for dementia evaluation (both for initial and follow‐up visits), status and main purpose of neuropsychological testing, and neuropsychological assessment tools used in routine clinical practice. The respondents' answers were compiled and analyzed. Statistical analysis was performed using jmp 14.0 (SAS Institute, Cary, NC, USA) to analyze the differences between groups using the χ^2^‐test.

## Results

### Questionnaire response rate

A total of 75 of the questionnaires sent were reported as undeliverable, resulting in a total of 1783 eligible participants. The number of responses received, including both paper and Web form responses, was 574, yielding a questionnaire response rate of 32.2%. Additionally, 20 respondents (3.5%) stated that they found it challenging to answer the questionnaire, because they lacked detailed knowledge on neuropsychological testing; they were excluded. Finally, 554 respondents were included in the analysis.

### Survey results

#### Institutional affiliation

The respondents' affiliations included general hospitals (36%), medical centers for dementia (25%), clinics (21%) and special‐function hospitals (15%). Furthermore, 37%, 37%, 12%, 11% and 3% of the respondents belonged to the neurology, psychiatry/psychosomatic medicine, internal medicine/geriatrics, neurosurgery and rehabilitation medicine departments, respectively. The highest percentage of respondents (44%) had >20 years of experience in dementia care, followed by 10–15 years (24%), 15–20 years (19%), 5–10 years (12%) and <5 years (1%) of experience.

#### Dementia practice and evaluation

**Figure 1:**
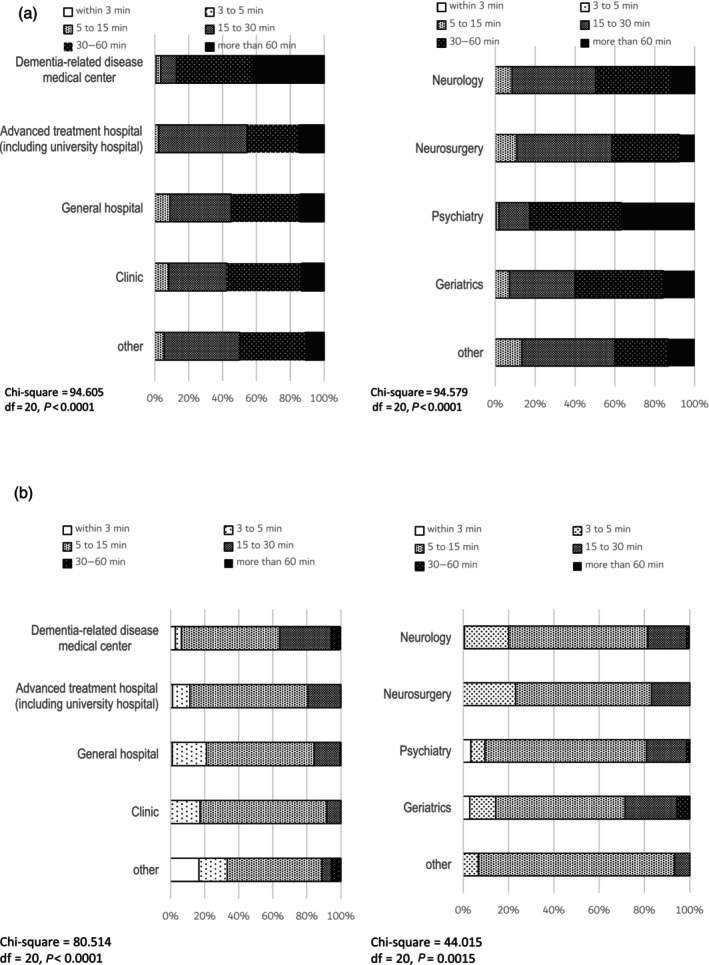
(a) Time spent on dementia evaluation at the initial visit. Most respondents indicated that the time required for dementia evaluation at the initial visit was 30–60 min, with approximately 20% of the respondents spending >60 min. The time spent on dementia evaluation tended to be longer at medical centers for dementia, psychiatry/psychosomatic medicine departments, and internal medicine/geriatrics departments than at other institutions and departments. (b) Time spent on dementia evaluation at the follow‐up visit. A few respondents spent >30 min on dementia evaluation during the follow‐up visit.

Most respondents reported that the time required for dementia evaluation was 30–60 min for an initial visit, and 5–15 min for a follow‐up visit (Fig. 1). Approximately 20% of the respondents spent >60 min on the initial consultation, but only a few spent >30 min on the follow‐up consultation. The time required for initial and follow‐up consultation showed significant differences both among different institutions and among different departments (Fig. 1a,b). Specifically, there was a tendency for longer consultation times in dementia‐related disease medical centers, psychiatry/psychosomatic medicine departments and internal medicine/geriatrics departments.

**Figure 2:**
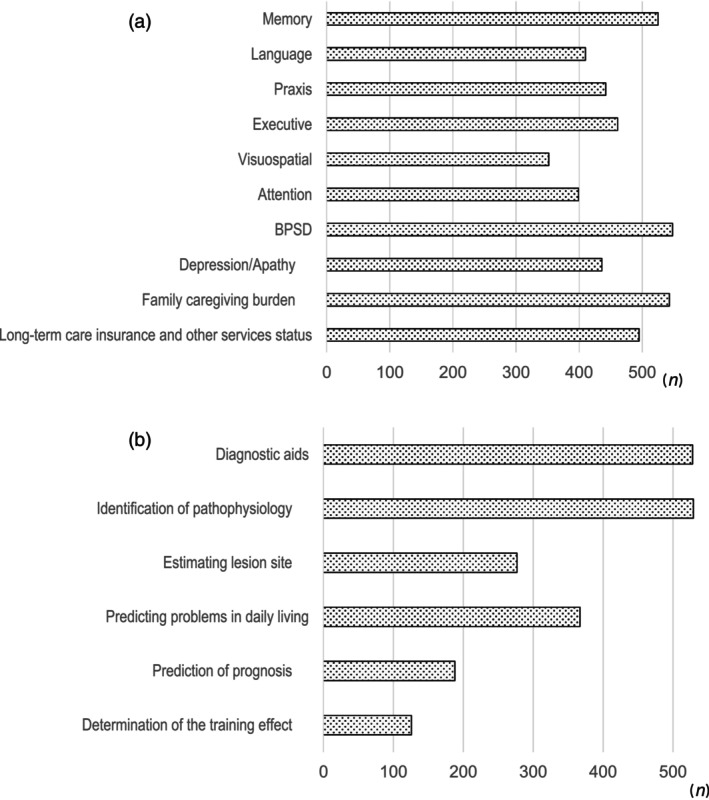
(a) Assessments considered important for dementia evaluation. Almost all respondents indicated that behavioral and psychological symptoms of dementia (BPSD) and family and caregiver burden are important aspects of dementia evaluation. (b) Primary purpose of neuropsychological testing. Over 90% of the respondents indicated that the main purposes of neuropsychological testing were to aid diagnosis and identify the pathophysiology.

Nearly all respondents considered behavioral and psychological symptoms of dementia (548 respondents), and family and caregiver burden (543 respondents) to be important aspects of dementia care. Memory (525 respondents), utilization of public services, such as long‐term care insurance (495 respondents), executive function (461 respondents), behavior (442 respondents), depression and apathy (436 respondents), and language (410 respondents), were considered important by >70% of the respondents (Fig. 2a). Over 90% of the respondents reported that the main purposes of neuropsychological testing were to aid diagnosis and identify the pathophysiology. Other purposes included the prediction of problems in daily living and estimation of the lesion site. A few respondents mentioned that they carried out neuropsychological testing to determine the training effect (Fig. 2b).

**Figure 3:**
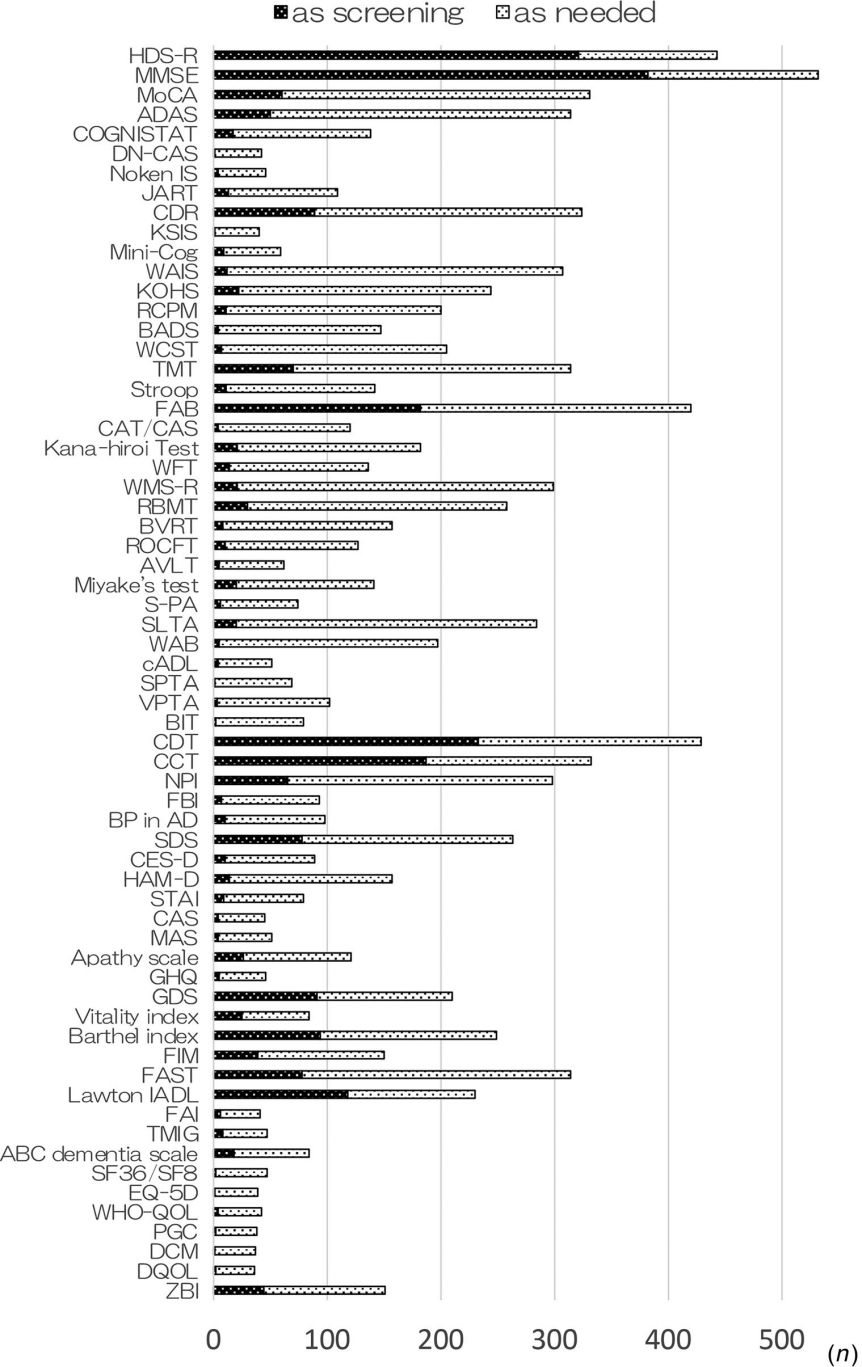
Neuropsychological assessments for dementia evaluation performed in clinical settings. The most commonly used screening tools for evaluating intelligence and mental functioning were the Hasegawa Dementia Scale‐Revised (HDS‐R) and Mini‐Mental State Examination (MMSE). The Clock Drawing Test (CDT), Cube Copying Test (CCT), and Frontal Assessment Battery (FAB) were also frequently used for screening. We surveyed dementia specialists in Japan to identify the assessment tools they use in clinical practice and related factors such as assessors and time required for each assessment. Our findings hold the potential to drive changes in the selection of screening and detailed assessment tools for treatment planning. ADAS, Alzheimer's Disease Assessment Scale; AVLT, Auditory Verbal Learning Test; BADS, Bevioural Assessment of the Dysexecutive Syndrome; BIT, Behavioural Inattention Test; BP in AD, Behavioral Pathology in Alzheimer's Disease Rating Scale; BVRT, Benton Visual Retention Test; cADL, Communication ADL; CAS, Cattele Anxiety Scale; CAT/CAS, Clinical Assessment for Attention/ Clinical Assessment for Spontaneity; CCT, Cube Copying Test; CDR, Clinical Dementia Rating; CDT, Clock Drawing Test; CES‐D, Center for Epidemiologic Studies Depression Scale; DCM, Dementia Care Mapping; DN‐CAS, Das‐Naglieri Cognitive Assessment System; DQOL, Dementia Quality Of Life instrument; EQ‐5D, EuroQol 5 dimensions; FAB, Frontal Assessment Battery; FAI, Frenchay Activities Index; FAST, Functional Assessment Staging of Alzheimer's Disease; FBI, Frontal Behavioral Inventory; FIM, Functional Independence Measure; GDS, Geriatric Depression Scale; GHQ, General Health Questionnaire; HAM‐D, Hamilton Depression Rating Scale; HDS‐R, Revised Hasegawa's Dementia Scale; JART, Japanese Adult Reading Test; KOHS, Kohs Cube Copying Test; KSIS, Kokuritsu‐Seiken Scale; Lawton IADL, Lawton Instrumental ADL; MAS, Manifest Anxiety Scale; Miyake's Test, Miyake's Paired Associate Learning Test; MMSE, Mini‐Mental State Examination; MoCA, Montreal Cognitive Assessment; Noken IC, Nouken Intelligence Scale; NPI, Neuropsychiatric Inventory; PGC, Philadelphia Geriatric Center Morale Scale; RBMT, Rivermead Memory Test; RCPM, Raven's Colored Progressive Matrices; ROCFT, Rey Osterrrieth Complex Figure Test; SDS, Self‐rating Depression Scale; SF36/SF8, MOS Short‐Form 36‐Item Health Survey; SLTA, Standard Language Test for Aphasia; S‐PA, Standard Verbal Paired Associate Learning Test; SPTA, Standard Performance Test for Apraxia; STAI, State Trait Anxiety Inventory; Stroop, Stroop Test; The abbreviations below are explained; TMG: Tokyo metropolitan institute of gerontology index of competence; TMT, Trail Making Test; VPTA, Visual Perception Test for Agnosia; WAB, Western Aphasia Battery; WAIS, Wechsler Adult Intelligence Scale; WCST, Winsconsin Card Sorting Test; WFT, Word Fluency Test; WHO‐QOL, World Health Organization Quality of Life; WMS‐R, Wechsler Memory Scale Revised; ZBI; Zarit caregiver Burden Interview.

The neuropsychological assessments used by the respondents in routine clinical practice are shown in Figure 3. The Hasegawa Dementia Scale‐Revised (HDS‐R) and Mini‐Mental State Examination (MMSE) were the most commonly used tests for intelligence and general mental function, and they were primarily used for screening. The Montreal Cognitive Assessment, Alzheimer's Disease Assessment Scale‐Cognitive Subscale, Clinical Dementia Rating and Wechsler Adult Intelligence Scale were also frequently used, but not primarily as screening instruments. The Revised Wechsler Memory Scale and Rivermead Behavioral Memory Test were used for memory assessment, although very few responders used them as screening tools. The Frontal Assessment Battery was frequently used to assess frontal lobe function, and approximately half of the time, it was used as a screening tool. The Wisconsin Card Sorting Test and Trail Making Test were used as required. The Clock Drawing Test (CDT) and Cube Copying Test (CCT) were commonly used for assessing visuospatial ability, and approximately half of the time, they were used as screening tools. Although social behavioral disorders and psychiatric conditions were not commonly assessed, screening was occasionally carried out using the Neuropsychiatric Inventory, Self‐Rating Depression Scale and Geriatric Depression Scale. The ability to carry out ADLs and instrumental ADLs was not extensively assessed; however, some respondents used the Barthel Index and Functional Independence Measure to evaluate this. The Lawton Instrumental ADL Scale and Functional Assessment Staging of Alzheimer's Disease tool were also used. Quality of life was infrequently assessed.

**Figure 4:**
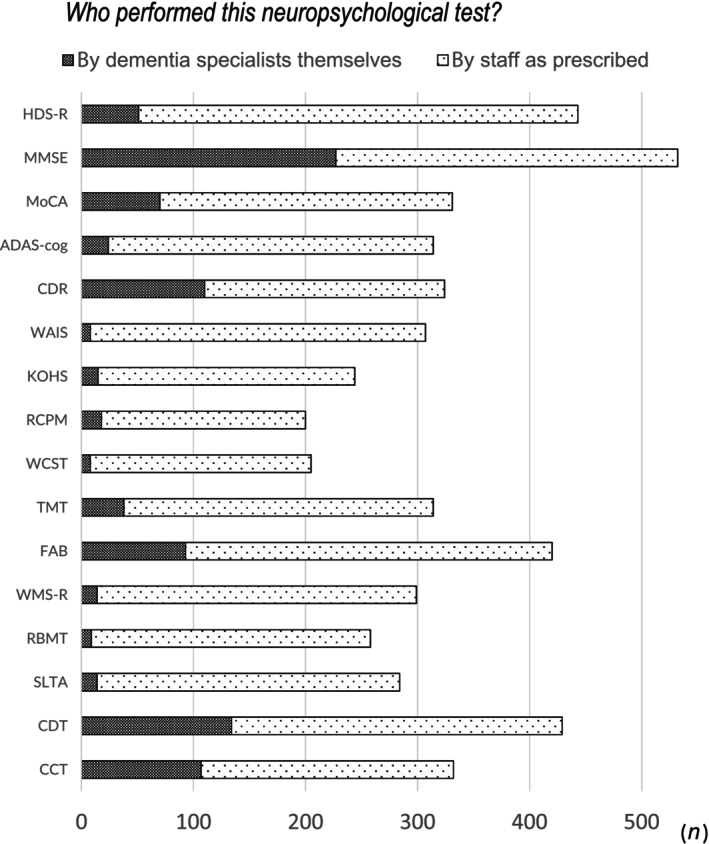
Person conducting the neuropsychological tests. The Mini‐Mental State Examination (MMSE) was conducted by the specialists themselves approximately half of the time, whereas the Clock Drawing Test (CDT) and Cube Copying Test (CCT) were performed by the specialists themselves approximately 30% of the time. However, most tests, including the Hasegawa Dementia Scale‐Revised (HDS‐R), were conducted by paramedical staff. ADAS‐cog, Alzheimer's Disease AssessmentScale; CDR, Clinical Dementia Rating; KOHS, Kohs Cube Copying Test; KSIS, Kokuritsu‐Seiken Scale; MoCA, Montreal Cognitive Assessment; RBMT, Rivermead Memory Test; RCPM, Raven's Colored Progressive Matrices; SLTA, Standard Language Test for Aphasia; TMT, Trail Making Test; WAIS, Wechsler Adult Intelligence Scale; WCST, Winsconsin Card Sorting Test; WMS‐R, Wechsler Memory Scale Revised.

**Figure 5:**
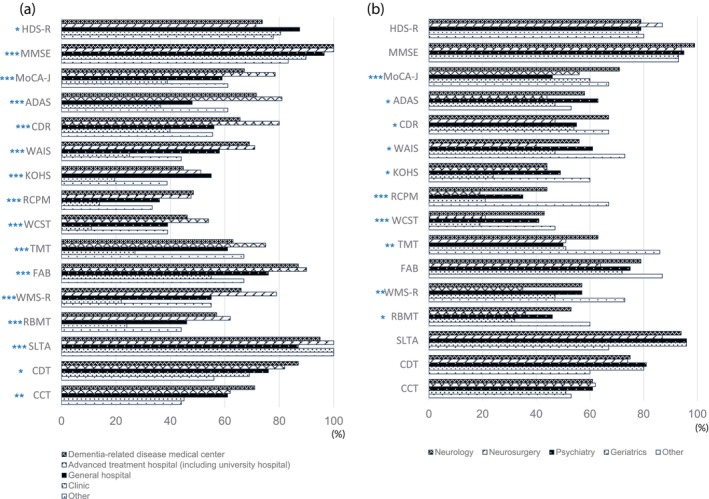
(a) Percentage of neuropsychological tests and facility. It shows the percentage of neuropsychological tests administered by each facility, and reveals significant differences in usage rates. χ^2^‐test *P < 0.01, **P < 0.001, ***P < 0.0001. (b) Percentage of neuropsychological tests by medical specialities. It shows the percentage of neuropsychological tests administered across departments and indicates that there is variation in usage except for the HDS‐R, MMSE, CDT, and CCT, which showed consistent percentages across departments. χ^2^‐test *P < 0.01, **P < 0.001, ***P < 0.0001. ADAS‐cog, Alzheimer's Disease AssessmentScale; CDR, Clinical Dementia Rating; KOHS, Kohs Cube Copying Test; KSIS, Kokuritsu‐Seiken Scale; MoCA, Montreal Cognitive Assessment; RBMT, Rivermead Memory Test; RCPM, Raven's Colored Progressive Matrices; SLTA, Standard Language Test for Aphasia; TMT, Trail Making Test; WAIS, Wechsler Adult Intelligence Scale; WCST, Winsconsin Card Sorting Test; WMS‐R, Wechsler Memory Scale Revised.

Figure 4 shows the neuropsychological tests used by >200 respondents and indicates who carried out these tests. Approximately half of the respondents stated that they administered the MMSE themselves, whereas approximately 30% administered the CDT and CCT themselves. In contrast, most neuropsychological tests, including the HDS‐R, were administered by paramedical staff (Fig. 4). In addition, we examined whether these extracted neuropsychological tests differed by the facility and department in which they worked (Fig. 5a,b). The results showed clear differences in the rates of implementation of any of the neuropsychological tests between facilities. We also found differences between the rates of many neuropsychological tests and the departments in which they were administered. However, no differences were found between the rates of HDS‐R, MMSE, CDT and CCT, and the departments. Although some responders commented in the free response section of the questionnaire that there was insufficient time for evaluation due to the large number of patients to be seen and that the reimbursement for evaluation is low, others expressed opinions that by carrying out thorough evaluations and medical interviews, they can provide care that is closely tailored to individuals with dementia and their families.

## Discussion

The demand for neuropsychological testing is increasing due to the increasing number of patients with dementia, higher brain dysfunction and developmental disorders.^9^ Neuropsychological assessment involves the evaluation of a patient's cognitive function based on their performance on various tests.^10^ Typically, a battery approach is used, in which multiple tests are carried out to assess different domains of cognitive function, including memory, attention, processing speed, reasoning, judgment, problem solving, spatial cognition, language, emotion, mood, motivation, attitude and self‐perception. Although these tests serve various purposes, such as diagnosing dementia and mild cognitive impairment, providing differential diagnoses, assessing the severity of the condition, evaluating the effect of treatment, and measuring outcomes in clinical trials, no single test can cover all aspects.^11^ In patients with dementia, functional decline is an important concern, making it particularly crucial to assess ADL ability and behavioral and psychological symptoms of dementia, and understand how they impact overall disability. However, dementia specialists' current understanding and practices regarding these assessments are not well established. To determine this, we carried out a questionnaire survey among dementia specialists.

According to a 2020 survey carried out by the Ministry of Health, Labor and Welfare in Japan, approximately 70% of general outpatient visits in hospitals lasted <10 min.^12^ In contrast, most initial consultations with dementia specialists took >15 min, with most consultations exceeding 30 min. Even for follow‐up visits, many respondents reported examination times >15 min. Although these results should be generalized with caution, owing to potential population biases, it is evident that dementia specialists show dedication in their practice. Despite the limited clinical hours, they utilize assessment tools, such as the HDS‐R and MMSE, for quickly screening general cognitive function. Additionally, they use other tools that enable brief assessment (e.g. Frontal Assessment Battery and Montreal Cognitive Assessment) or do not require specialized equipment (e.g. CDT and CCT).

As for evaluations during consultations, evaluation methods that can assess general cognitive functions, such as HDS‐R and MMSE, were basically caried out, whereas evaluation methods that can be carried out in a short time and with little detail (FAB, MoCA etc.) and evaluation methods that do not require special equipment (CDT, CCT) were carried out by the physician in charge themself. All of these evaluation methods can be carried out in approximately 10 min, and it was characteristic that among the many evaluation methods, those that can be carried out in a short time were selected regardless of the specialist's department. Approximately one‐third of physicians directly involved in dementia care carried out these screenings. In essence, physicians use practical tools that can be effectively implemented within the limited available time and that are reimbursable under insurance treatment, aiming to gain a broad understanding of the symptoms that can guide treatment selection.

Appropriate screening was carried out to assess general cognitive and mental function. However, only approximately one‐third to half of the respondents conducted specific assessments for individual cognitive domains, such as memory, which is impaired in many individuals with dementia, and is considered a crucial component of dementia practice and evaluation. It is possible that they are using the simple memory assessments included in the HDS‐R and MMSE instead. Similar patterns were observed for other cognitive domains. Although specialized evaluations covering many domains are necessary for identifying the pathophysiology and to aid diagnosis, which >90% of respondents consider the primary purposes of assessment, it is apparent that such detailed evaluations are not being carried out in practice. This highlights the need to educate physicians engaged in dementia practice, and determine the importance and necessity of comprehensive assessments that align with the physicians' goals. Other issues raised include limited manpower for evaluation, problems with reimbursement (insurance points) and a lack of evaluator skills. Further research is required to address the gaps in the medical system, practice framework, and professional healthcare education related to dementia practice and evaluation.

Furthermore, approximately half of the respondents of our survey carried out assessments related to daily life, and very few assessments related to quality of life were performed. However, the accurate assessment, improvement and maintenance of functional abilities in patients with dementia is important. This necessitates a comprehensive evaluation of social factors, such as ADL ability, quality of life, nursing care and residential environment, in addition to diagnosis and treatment.^6^, ^13^ Medical professionals specializing in dementia management should systematically and comprehensively evaluate the clinical and lifestyle profiles of their patients, and this cannot be done through neuropsychological testing alone. However, time‐consuming assessment tools are rarely used in routine clinical practice, even if they offer the benefit of a detailed assessment. Therefore, when selecting a standardized assessment tool, it is essential to consider comprehensive tools that can be administered quickly in clinical settings. It is also necessary to distinguish between simple screening tools that assess multiple domains and detailed assessment tools used to inform treatment and care, using the best tool depending on the purpose of evaluation.

One limitation of the present study is that the response rate was 32.2%, indicating the potential for selection bias. The characteristics of the non‐respondents remain unknown, and their perspectives on neuropsychological testing might differ from those of the respondents. Consequently, caution must be exercised when generalizing the present findings to the entire population of healthcare professionals involved in dementia care.

Finally, the HDS‐R, a screening assessment of cognitive function, has long been used in Japanese medical practice and is still widely used. However, dementia is a global disease, and Japan, being at the forefront of geriatric medicine, is in a position to provide information on symptoms, pathophysiology, and treatment effects to a wide range of foreign countries and to disseminate medical information. For this reason, it was considered necessary to select an evaluation method that is not only the HDS‐R, but also the MMSE, MoCA and other internationally used evaluation methods, with a view to international comparisons. It is necessary to raise awareness of this issue domestically, as well as internationally; select assessment methods that can be widely used internationally, not only those unique to each country; and make efforts to establish a global standard for dementia assessment.

## Disclosure statement

The authors declare no conflict of interest.

## Data Availability

The data are not publicly available due to privacy or ethical restrictions.
